# Protective Effects of Casticin From *Vitex trifolia* Alleviate Eosinophilic Airway Inflammation and Oxidative Stress in a Murine Asthma Model

**DOI:** 10.3389/fphar.2018.00635

**Published:** 2018-06-14

**Authors:** Chian-Jiun Liou, Ching-Yi Cheng, Kuo-Wei Yeh, Yi-Hong Wu, Wen-Chung Huang

**Affiliations:** ^1^Division of Basic Medical Sciences, Department of Nursing, Research Center for Chinese Herbal Medicine, Graduate Institute of Health Industry Technology, Chang Gung University of Science and Technology, Taoyuan City, Taiwan; ^2^Division of Allergy, Asthma, and Rheumatology, Department of Pediatrics, Chang Gung Memorial Hospital, Taoyuan City, Taiwan; ^3^Graduate Institute of Health Industry Technology, Research Center for Food and Cosmetic Safety, Research Center for Chinese Herbal Medicine, College of Human Ecology, Chang Gung University of Science and Technology, Taoyuan City, Taiwan; ^4^Department of Ophthalmology, Chang Gung Memorial Hospital, Taoyuan City, Taiwan; ^5^Department of Traditional Chinese Medicine, Chang Gung Memorial Hospital, Keelung, Taiwan; ^6^Division of Chinese Internal Medicine, Center for Traditional Chinese Medicine, Chang Gung Memorial Hospital, Taoyuan City, Taiwan; ^7^School of Traditional Chinese Medicine, College of Medicine, Chang Gung University, Taoyuan City, Taiwan

**Keywords:** asthma, casticin, cytokine, eosinophil, oxidative stress

## Abstract

Casticin has been isolated from *Vitex trifolia* and found to have anti-inflammatory and anti-tumor properties. We also previously discovered that casticin can reduce pro-inflammatory cytokines and ICAM-1 expression in inflammatory pulmonary epithelial cells. In the present study, we evaluated whether casticin reduced airway hyper-responsiveness (AHR), airway inflammation, and oxidative stress in the lungs of a murine asthma model and alleviated inflammatory and oxidative responses in tracheal epithelial cells. Female BALB/c mice were randomly divided into five groups: normal controls, ovalbumin (OVA)-induced asthma, and OVA-induced asthma treated with intraperitoneal injection of casticin (5 or 10 mg/kg) or prednisolone (5 mg/kg). Casticin reduced AHR, goblet cell hyperplasia, and oxidative responses in the lungs of mice with asthma. Mechanistic studies revealed that casticin attenuated the levels of Th2 cytokine in bronchoalveolar lavage fluids and regulated the expression of Th2 cytokine and chemokine genes in the lung. Casticin also significantly regulated oxidative stress and reduced inflammation in the lungs of mice with asthma. Consequently, inflammatory tracheal epithelial BEAS-2B cells treated with casticin had significantly suppressed levels of pro-inflammatory cytokines and eotaxin, and reduced THP-1 monocyte cell adherence to BEAS-2B cells via suppressed ICAM-1 expression. Thus, casticin is a powerful immunomodulator, ameliorating pathological changes by suppressing Th2 cytokine expression in mice with asthma.

## Introduction

Asthma is a common allergic and inflammatory disease of the respiratory system. Due to worsening air pollution and allergy causing immune system dysfunction, the prevalence of asthma has increased worldwide (DeKruyff et al., 2014; Rice et al., 2018). Approximately, 300 million people have symptoms of asthma, which is a great burden to global medical resources (To et al., 2012). Pathological features of asthma include chest tightness, coughing, and shortness of breath (Tanno et al., 2017). During an acute asthma attack, smooth muscle contraction exaggerates airway narrowing, and allergy-stimulated respiratory epithelial cells induce excess mucus secretion to block airways, resulting in breathing difficulties and potentially even suffocation (Lambrecht and Hammad, 2012).

Recent studies have confirmed immune system imbalance as one of the important factors in allergic asthma (Duong et al., 2015). In asthma patients, activated Th2 cells release excess cytokines to stimulate airway hyper-responsiveness (AHR) and induce eosinophil infiltration, resulting in exacerbated inflammation and allergic reaction in the lungs (Parulekar et al., 2016). Furthermore, Th2-associated cytokines induce goblet cell hyperplasia and mucus secretion, causing severe respiratory obstruction, and irritated oxidative responses that contribute to lung damage in mice with asthma. Therefore, regulating the immune system and attenuating the improper activation of Th2 cells is important for the amelioration of asthma (Steinke and Lawrence, 2014).

Pro-inflammatory cytokines secreted by Th2 cells not only induce inflammation and the activation of eosinophils, monocytes, and neutrophils in the lungs, but also stimulate airway epithelial cells to secrete more cytokines and chemokines, exacerbating the inflammatory reaction and cell damage in the airways and lungs, reducing pulmonary function (Lambrecht and Hammad, 2012; Leomicronn, 2017). Specifically, inflamed respiratory epithelial cells induce excessive reactive oxygen species (ROS) that deteriorate AHR and increase the smooth muscle thickness, narrowing the airway (Nesi et al., 2017a). Furthermore, the activated eosinophils would release more eosinophil peroxidase, which could catalyze hydrogen peroxide and chloride ions to form hypochlorous acid, causing oxidative stress and damaging the lung tissue (Patel and Sur, 2017). Therefore, suppressing the activation of Th2 cells would reduce inflammatory and oxidative damage of the airways and improve asthma symptoms.

*Vitex trifolia* L. is a species of Verbenaceae distributed throughout China and other East Asian areas, and its fruit has been used to treat headache, gingival swelling, inflammation, and dizziness (Matsui et al., 2012). *V. trifolia* is also used to treat cancer in Sichuan, China (Matsui et al., 2009). Casticin has been isolated from *V. trifolia* or *Vitex rotundifolia* and reported to have anti-cancer effects, inhibit inflammation in LPS-induced acute lung injury in mice, and improve the cigarette smoke-induced acute lung inflammatory response in mice (Lee et al., 2015; Wang et al., 2016; Chou et al., 2018). We previously found that casticin can reduce pro-inflammatory cytokine and ICAM-1 expression by blocking the NF-κB, MAPK, and PI3K/Akt pathways in IL-1β-activated A549 human lung epithelial cells (Liou et al., 2014; Liou and Huang, 2017). Therefore, we speculated that casticin may improve asthma by suppressing inflammation and oxidative stress. In the current study, asthmatic mice were treated with casticin by intraperitoneal injection to evaluate whether casticin ameliorates asthma symptoms. We also investigated the effect of casticin on regulated immune function, oxidative stress, and inflammation in the murine model of asthma.

## Materials and Methods

### Animals

Female BALB/c mice (6 to 8 weeks old, 20–25 g) were purchased from the National Laboratory Animal Center in Taiwan. All mice were housed in an air-conventional animal house at a controlled room temperature (22–24°C) with a 12 h natural light/dark cycle. Water and standard chow diet were provided *ad libitum*. Animal experiment and care procedures were approved by the Laboratory Animal Care Committee of Chang Gung University of Science and Technology (IACUC Approval No. 2016-004).

### Drug Treatment and Sensitization

**FIGURE 1:**
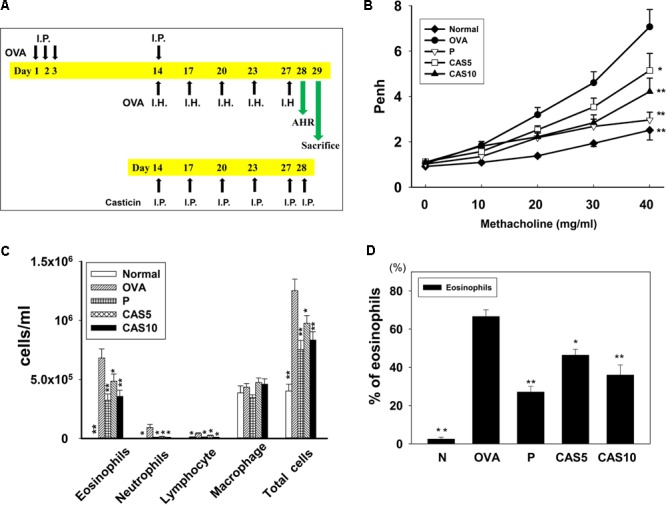
Effect of casticin on AHR and cell counts in BALF from asthmatic mice. **(A)** On days 1–3 and 14, mice sensitized with OVA by intraperitoneal injection (I.P.) and challenged with 2% OVA inhalation (I.H.) on days 14, 17, 20, 23, and 27. One hour before the OVA challenge or methacholine inhalation, mice were treated with I.P. casticin or prednisolone. **(B)** AHR (Penh values) was measured via inhalation of increasing methacholine doses (0–40 mg/ml). **(C)** Inflammatory cells and total cells were measured in BALF and **(D)** presented the percentage of eosinophils in BALF. The data are presented as means ± SEM of three independent experiments (*n* = 12). ^∗^*p* < 0.05, ^∗∗^*p* < 0.01 compared to the OVA control group. Mice were divided into normal (N), OVA-sensitized mice (OVA), prednisolone control (P), and casticin treatment (CAS5 and CAS10) groups.

Casticin (isolated from *V. trifolia*, ≥98% purity by HPLC; Sigma-Aldrich, St. Louis, MO, United States) was dissolved in DMSO (100%). The mice were sensitized to the experimental protocol as shown in **Figure 1A**. Briefly, on days 1–3 and 14, the mice sensitized with 50 μg ovalbumin (OVA; Sigma) mixed with 0.8 mg aluminum hydroxide (Thermo, Rockford, IL, United States) in 200 μl normal saline by intraperitoneal injection. Mice were challenged with inhalation of 2% OVA using an ultrasonic nebulizer (DeVilbiss Pulmo-Aide 5650D, United States) for 30 min on days 14, 17, 20, 23, and 27. The mice were treated with casticin, prednisolone, or normal saline by intraperitoneal injection 1 h before the OVA challenge or methacholine inhalation. AHR was detected on day 28 and the mice sacrificed to evaluate immune, inflammatory, and asthma pathology on day 29. All mice were randomly subdivided into five groups (*n* = 12 each): normal (N group), healthy mice sensitized with normal saline and received 50 μl DMSO (100%) by intraperitoneal injection; OVA-sensitized mice (OVA group), mice sensitized with OVA and received 50 μl DMSO by intraperitoneal injection; prednisolone control (P group), mice sensitized with OVA and received 5 mg/kg prednisolone (dissolved in 50 μl 100% DMSO) by intraperitoneal injection; and OVA-sensitized mice treated with 5 or 10 mg/kg casticin (dissolved in 50 μl 100% DMSO) by intraperitoneal injection (CAS5 and CAS10 groups, respectively).

### Evaluation of AHR

Twenty-four hours after the last challenge, AHR was measured to evaluate airway function as described previously (Huang et al., 2017). All mice inhaled aerosolized methacholine (0 to 40 mg/ml) for 3 min before being placed in a chamber to record the enhanced pause (Penh) for measurement of AHR using whole-body plethysmograph (Buxco Electronics, Troy, NY, United States).

### Histopathological Evaluation of Lung Tissue

Mouse lung tissues were removed and fixed with 10% formalin, and then embedded in paraffin and sectioned (6-μm-thick) for staining as described previously (Liou et al., 2016). Tissue sections were stained with hematoxylin and eosin (H&E) to observe eosinophil infiltration or periodic acid-Schiff (PAS; Sigma) to evaluate goblet cell hyperplasia.

### Inflammatory Cells in Bronchoalveolar Lavage Fluid

Mice were anesthetized with 4% isoflurane and bronchoalveolar lavage fluid (BALF) collected as described previously (Kuo et al., 2017). Briefly, the mice were incubated with an indwelling needle into the trachea, the lungs washed three times with 1 ml normal saline, and supernatant collected to detect the levels of cytokines and chemokines. After a cytospin centrifugation, Giemsa stain (Sigma) was used to count the cells and evaluate cell morphology. Furthermore, the percentages of eosinophils obtained using the cell counts in 500 BALF cells.

### Glutathione (GSH) Assay

A Glutathione Assay Kit (Sigma) was used to investigate the levels of glutathione (glutathione disulfide, reduced glutathione, and total glutathione) in lung tissues according to the manufacturer’s instructions. Briefly, 50 mg lung tissues were kept on 5% 5-sulfosalicylic acid solution and homogenized by homogenizer (FastPrep-24, MP Biomedicals, Santa Ana, CA, United States). The sample centrifuged at 10,000 *g* for 10 min, and supernatant collected to detect the glutathione levels as the optical density at 412 nm using a microplate reader (Multiskan FC, Thermo, Waltham, MA, United States).

### Malondialdehyde (MDA) Activity

To detect malondialdehyde (MDA) activity in lung tissues, we using a Lipid Peroxidation Assay Kit (Sigma) according to the manufacturer’s instructions. Briefly, 10 mg lung tissues were homogenized in 300 μl of the lysis buffer containing 3 μl butylated hydroxy toluene.

The sample centrifuged at 13,000 *g* for 10 min, and supernatant added perchloric acid for protein precipitation. Next, the sample centrifuged and supernatant collected to detect MDA activity using a multi-mode microplate reader (BioTek SynergyHT, Bedfordshire, United Kingdom).

### Serum Collection and Splenocyte Culture

Mice were anesthetized and blood harvested via the orbital vascular plexus and centrifuged at 6,000 rpm for 5 min. The serum was obtained to detect OVA-specific antibodies. In addition, splenocytes (5 × 10^6^ cells/ml) were cultured in RPMI 1640 medium containing 100 μg/ml OVA for five continuous days and cytokines measured as described previously (Kuo et al., 2017).

### Enzyme-Linked Immunosorbent Assay

Bronchoalveolar lavage fluid and cell culture media underwent enzyme-linked immunosorbent assay (ELISA) to detect the levels of cytokines and chemokines as described previously (Huang et al., 2017). ELISA kits were used for tumor necrosis factor-α (TNF-α), interferon-γ (IFN-γ), MCP-1, CCL5, CCL11, CCL24, CCL26, IL-13, IL-8, IL-6, IL-5, IL-4, and intercellular adhesion molecule 1 (ICAM-1) (R&D Systems, Minneapolis, MN, United States) according to the manufacturer’s instructions. Serum OVA-specific antibodies, including OVA-IgE and OVA-IgG1, were assayed using a specific ELISA kit (BD Biosciences, Franklin Lakes, NJ, United States) as described previously (Kuo et al., 2017). Serum was diluted fivefold to measure the OVA-IgE absorbance values as the optical density at 450 nm using a microplate reader (Thermo). The units of OVA-IgG1 were defined by comparing the OD_450_ values to make a standard curve using pooled serum.

### Western Blot

Mouse lungs were homogenized and lysed using RIPA buffer containing protease inhibitors (Sigma), and equal amounts of lung protein separated by 10% SDS polyacrylamide gel electrophoresis (PAGE). The proteins were transferred onto polyvinylidene fluoride (PVDF) membranes (Millipore, Billerica, MA, United States), which were incubated overnight with primary antibodies. Subsequently, the PVDF membranes were incubated with secondary antibodies (1:5000) and specific proteins expressed using Luminol/Enhancer Solution (Millipore) in a BioSpectrum 600 system (UVP, Upland, CA, United States). Primary antibodies included COX-2 (1:1000), HO-1 (1:1000), iNOS (1:1000), Nrf2 (1:1000), Lamin B1 (1:2000) (Santa Cruz, CA, United States), and β-actin (1:5000) (Sigma).

### RNA Isolation and Quantitative Real-Time PCR Analysis

RNA was extracted from lung tissues using TRIzol reagent (Life Technologies, Carlsbad, CA, United States) and reverse-transcribed to acquire cDNA using a cDNA synthesis kit (Life Technologies). Quantitative real-time PCR using SYBR Green Master Mix (Bio-Rad, San Francisco, CA, United States) was performed using a spectrofluorometric thermal cycler (iCycler; Bio-Rad, San Francisco, CA, United States) to evaluate the expression of specific genes as described previously (Huang et al., 2017). The cycling conditions were as follows: samples preincubated at 95°C for 10 min. Next, the PCR was performed as 40 cycles of 95°C for 15 s and 60°C for 1 min. **Table 1** lists the primers used for PCR.

**Table 1:** Primers used in real-time PCR analyses of cytokine and chemokine mRNA expression.

Gene	Forward primer	Reverse primer
IL-6	AGGACCAAGACCATCCAATTCA	GCTTAGGCATAACGCACTAGG
CCL11	GGCTTCATGTAGTTCCAGAT	CCATTGTGTTCCTCAATAATCC
CCL24	AGGCAGTGAGAACCAAGT	GCGTCAATACCTATGTCCAA
COX-2	ACCAGCAGTTCCAGTATCAGA	CAGGAGGATGGAGTTGTTGTAG
iNOS	TTCCACAACCACCTCAAGCA	TTAAGGCATCACAGTCCGAGTC
IFN-γ	CAGCAACAACATAAGCGTCATT	ACCTCAAACTTGGCAATACTCA
IL-4	TCCGTGCTTGAAGAAGAACTC	GTGATGTGGACTTGGACTCATT
IL-5	ATCCTCCTGCCTCCTCTTCC	GGTTCCATCTCCAGCACTTCA
IL-13	GCTCCAGCATTGAAGCAGTG	CGTGGCAGACAGGAGTGTT
ICAM-1	AACAGAATGGTAGACAGCAT	TCCACCGAGTCCTCTTAG
Gob5	AATGGATGAATGGCTCAGTGAT	TATTGTAGGAGGATGCGTTGTC
MUC5AC	AATGCTGGTGCCTGTGTCT	CCTCCTATGCCATCTGTTGTG
β-Actin	AAGACCTCTATGCCAACACAGT	AGCCAGAGCAGTAATCTCCTTC

### BEAS-2B Cell Culture and Casticin Treatment

Human bronchial epithelial BEAS-2B cells (American Type Culture Collection, Manassas, VA, United States) were cultured in DMEM/F12 and seeded into 24-well plates. Casticin was dissolved in 100% DMSO at a concentration of 20 mM as a stock solution, and all experimental culture medium DMSO was ≤0.1%. BEAS-2B cells were pre-treated with casticin (5–20 μM) for 1 h, and then treated with 20 ng/ml IL-4 and 10 ng/ml TNF-α for 24 h, or 10 ng/ml TNF-α for 24 h. The media were used to detect cytokine or chemokine production using specific ELISA kits.

### ROS Production Analysis

Tumor necrosis factor α-induced BEAS-2B cells were seeded in 96-well plates and treated with various concentrations of casticin for 24 h as described previously (Huang et al., 2017). The cells were then incubated with 20 μM 2′,7′-dichlorofluorescin diacetate (DCFH-DA) for 30 min to analyze ROS expression using a Multi-Mode microplate reader (BioTek synergy HT). Intracellular ROS were also observed using a fluorescence microscope (Olympus, Tokyo, Japan).

### Cell–Cell Adhesion Analysis

Human bronchial epithelial BEAS-2B cells were cultured in DMEM/F12 and seeded into 6-well plates. BEAS-2B cells were pre-treated with casticin (5–20 μM) for 1 h, and then incubated with 10 ng/ml TNF-α for 24 h as described previously (Huang et al., 2016). Human monocytic THP-1 cells (5 × 10^6^/ml) (Bioresource Collection and Research Center, Taiwan) were incubated with calcein-AM solution (Sigma) for 0.5 h. Next, THP-1 cells were co-cultured with BEAS-2B cells and observed under a fluorescence microscope (Olympus).

### Statistical Analysis

Data are expressed as mean ± SEM of at least three independent experiments and evaluated by one-way analysis of variance (ANOVA) followed by Tukey–Kramer *post hoc* test. Significance was set at *p* < 0.05.

## Results

### Effect of Casticin on OVA-Induced AHR in Mice

Airway hyper-responsiveness was measured as Penh values in response to inhaling gradually increasing doses of methacholine (0–40 mg/ml) to evaluate whether casticin improves airway function in asthmatic mice. The Penh values significantly increased in OVA-sensitized mice compared to normal mice (**Figure 1B**). We found that a high inhaled dose of methacholine (40 mg/ml) in casticin or prednisolone-treated mice with asthma significantly decreased the Penh values compared to the OVA group (7.17 ± 0.71 vs. P: 2.96 ± 0.96, *p* < 0.01; CAS5: 5.14 ± 0.95, *p* < 0.05; and CAS10: 4.21 ± 0.72, *p* < 0.01). Thus, casticin could significantly suppress AHR in asthmatic mice.

### Effect of Casticin on Inflammatory Cells in BALF

Bronchoalveolar lavage fluid collected and Giemsa stain calculated the total cell count and cell morphology.

Compared to the OVA group, asthmatic mice treated with casticin or prednisolone had significantly reduced numbers of total cells (1.3 × 10^6^± 1.0 × 10^5^ vs. P: 7.5 × 10^5^± 7.7 × 10^4^, *p* < 0.01; CAS5: 9.7 × 10^5^± 6.4 × 10^4^, *p* < 0.05; and CAS10: 8.3 × 10^5^± 7.0 × 10^4^, *p* < 0.01) and eosinophils (6.8 × 10^5^± 7.6 × 10^4^ vs. P: 3.2 × 10^5^± 5.4 × 10^4^, *p* < 0.01; CAS5: 4.8 × 10^5^± 6.0 × 10^3^, *p* < 0.05; and CAS10: 3.6 × 10^5^± 5.2 × 10^4^, *p* < 0.01; **Figure 1C**). Casticin and prednisolone also significantly decreased the percentage of eosinophils in BALF compared to the OVA group (**Figure 1D**).

### Effect of Casticin on Goblet Cell Hyperplasia and Eosinophil Infiltration in the Lungs

**FIGURE 2:**
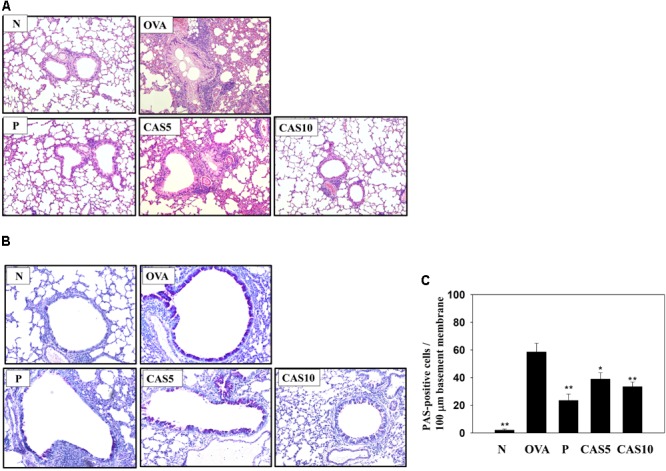
Effects of casticin (CAS) on asthmatic lung tissue. Histological sections of lung tissues from normal (N) and OVA-stimulated (OVA) mice with or without prednisolone (P) and CAS treatment. **(**A**)** CAS reduced eosinophil infiltration (H&E stain; 200× magnification). **(B)** Periodic acid-Schiff (PAS)-stained lung sections show goblet cell hyperplasia (200× magnification). **(C)** Results are expressed as the number of PAS-positive cells per 100 μm of basement membrane. The data are presented as means ± SEM of three independent experiments (*n* = 6). ^∗^*p* < 0.05, ^∗∗^*p* < 0.01 compared to the OVA control group.

H&E staining of eosinophil infiltration in the lungs showed that casticin reduced eosinophil infiltration between the bronchus and blood vessels of the lungs in asthmatic mice (**Figure 2A**). In the airways of asthma patients, allergens would stimulate goblet cell hyperplasia to secret excessive mucus (Sastre et al., 2017). Hence, we also evaluated goblet cell hyperplasia in the bronchus with PAS staining, demonstrating that casticin obviously reduced goblet cell hyperplasia compared to OVA-sensitized mice (**Figures 2B,C**). Prednisolone also clearly improved eosinophil infiltration and goblet cell hyperplasia in the lungs of asthmatic mice.

### Casticin Modulated Chemokine and Cytokine Production in BALF and Lung Tissue

**FIGURE 3:**
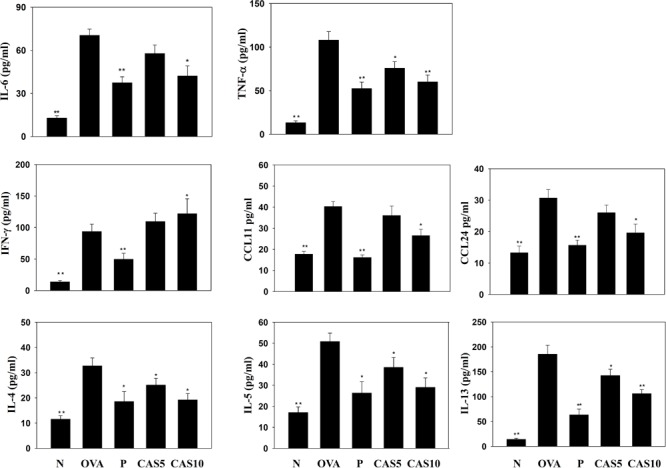
Effects of casticin on the levels of cytokines and chemokines in BALF. The concentrations of IL-6, TNF-α, IFN-γ, CCL11, CCL24, IL-4, IL-5, and IL-13 were measured by ELISA in BALF from normal (N) and OVA-stimulated (OVA) mice treated with or without casticin (CAS 5-10) and prednisolone (P). The data are presented as means ± SEM of three independent experiments (*n* = 8). ^∗^*p* < 0.05, ^∗∗^*p* < 0.01 compared to the OVA control group.

**FIGURE 4:**
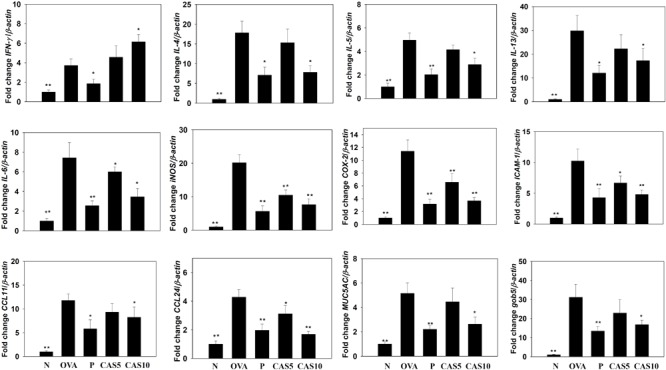
Effects of casticin (CAS) on the expression of chemokines, inflammatory mediators, and cytokines in the lungs. Gene expression was determined using real-time RT-PCR with lung tissues from normal (N) and OVA-stimulated (OVA) mice with or without CAS and prednisolone (P) treatment. Fold-changes in expression were measured relative to β-actin expression (internal control). The data are presented as means ± SEM of three independent experiments (*n* = 8). ^∗^*p* < 0.05, ^∗∗^*p* < 0.01 compared to OVA control mice.

Cytokines and chemokines in the BALF were detected using ELISA, showing that casticin significantly reduced IL-6 levels compared to the OVA group (70.4 ± 4.4 pg/ml vs. P: 37.4 ± 4.1 pg/ml, *p* < 0.01; CAS5: 57.7 ± 5.9 pg/ml, *p* = 0.21; and CAS10: 42.1 ± 7.1 pg/ml, *p* < 0.05; **Figure 3**). However, casticin clearly increased INF-γ levels compared to the OVA group (93.6 ± 11.7 pg/ml vs. P: 49.6 ± 9.5 pg/ml, *p* < 0.01; CAS5: 109.5 ± 9.5 pg/ml, *p* = 0.38; and CAS10: 121.9 ± 23.7 pg/ml, *p* < 0.05). Moreover, the casticin and prednisolone groups had significantly reduced TNF-α, IL-4, IL-5, IL-13, CCL11, and CCL24 production compared to the OVA group. In the lung tissue demonstrated, casticin significantly attenuated IL-4, IL-5, IL-6, IL-13, iNOS, COX-2, ICAM-1, CCL11, CCL24, MUC5AC, and Gob5 gene expression compared to OVA-sensitized mice, and promoted expression of the IFN-γ gene compared to asthmatic mice (**Figure 4**).

### Casticin Regulated Inflammatory Mediators and Oxidative Stress in Lung Tissue

**FIGURE 5:**
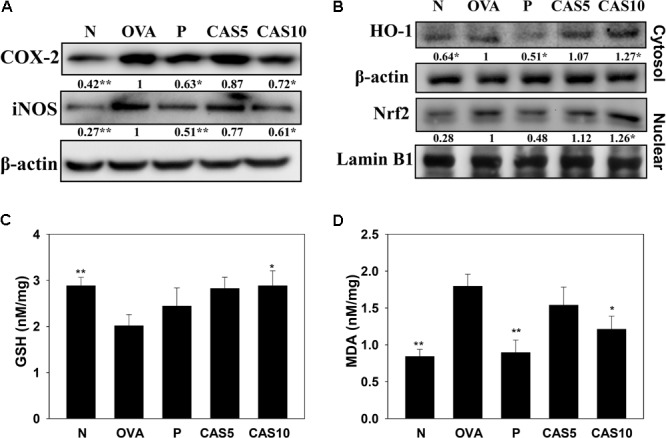
Effects of casticin (CAS) on inflammatory mediators and oxidative stress factors. **(A)** Western blot showing that CAS suppressed COX-2 and iNOS expression, and **(B)** regulated HO-1 and Nrf2 expression in lung tissue from normal (N) and OVA-stimulated (OVA) mice with or without (CAS) and prednisolone (P) treatment. **(C)** GSH activity and **(D)** MDA activity in the lung tissues of mice. The data are presented as means ± SEM of three independent experiments (*n* = 6). ^∗^*p* < 0.05, ^∗∗^*p* < 0.01 compared to OVA control mice.

Asthma attacks can induce inflammatory and oxidative stress, causing cell damage in the lung (Nesi et al., 2017b). In this study, casticin reduced iNOS and COX-2 production in the lungs compared to OVA-sensitized mice (**Figure 5A**). Previous studies demonstrated that anti-oxidant HO-1 can protect against and reduce oxidative stress in the lungs (Araujo et al., 2012). We found that casticin promotes HO-1 and increases nuclear Nrf2 expression in the lungs compared to OVA-sensitized mice (**Figure 5B**). Furthermore, casticin also obviously decreased MDA activity and increased GSH production in the lungs compared to the OVA group (**Figures 5C,D**).

### Casticin Modulated Serum OVA-Specific Antibody and Splenocyte Cytokine Production

**FIGURE 6:**
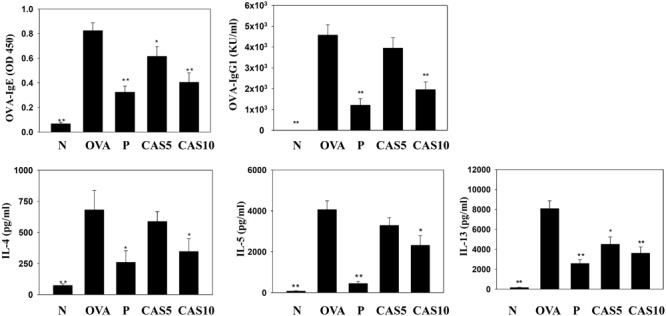
Effects of casticin (CAS) on OVA-specific antibodies in serum. Serum levels of OVA-IgE and OVA-IgG1 in normal (N) and OVA-stimulated (OVA) mice with or without CAS and prednisolone (P) treatment. CAS also changed the levels of IL-4, IL-5, and IL-13 produced by OVA-activated splenocytes. The data are presented as means ± SEM of three independent experiments (*n* = 12). ^∗^*p* < 0.05, ^∗∗^*p* < 0.01 compared to the OVA control group.

In the serum from OVA-sensitized mice, casticin significantly inhibited OVA-IgG1 and OVA-IgE. In the media from splenocyte culture, casticin also clearly suppressed IL-4, IL-5, and IL-13 production compared to OVA-sensitized mice (**Figure 6**).

### Casticin Reduced Inflammatory Mediators in BEAS-2B Cells

**FIGURE 7:**
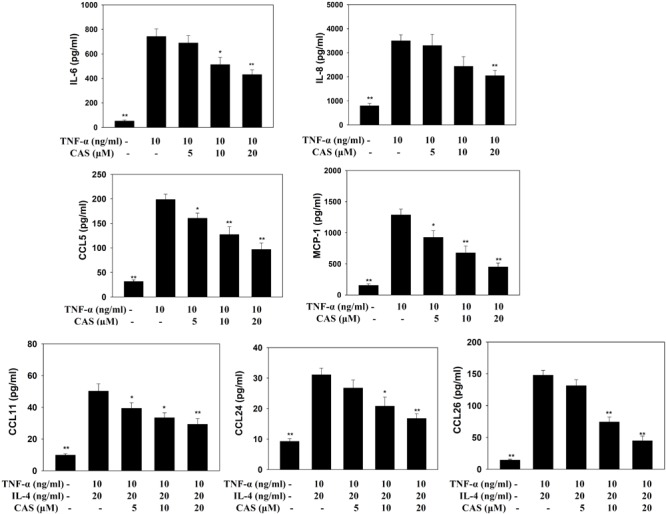
Effects of casticin (CAS) on cytokine and chemokine production in BEAS-2B cells. ELISA showing IL-6, IL-8, CCL5, MCP-1, CCL11, CCL24, and CCL26 levels in BAEAS-2B cells treated with CAS. The data are presented as means ± SEM of three independent experiments (*n* = 9). ^∗^*p* < 0.05, ^∗∗^*p* < 0.01 compared to BEAS-2B cells stimulated with TNF-α alone or TNF-α and IL-4.

In TNF-α-stimulated BEAS-2B cells, casticin reduced the levels of CCL5, MCP-1, IL-6, and IL-8. In BEAS-2B cells stimulated with IL-4 and TNF-α, casticin significantly attenuated the levels of CCL11, CCL24, and CCL26 (**Figure 7**).

### Casticin Inhibited Monocytic Cell Adherence to BEAS-2B Cells

**FIGURE 8:**
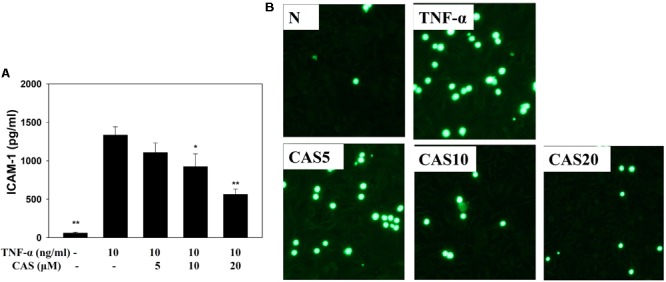
Casticin (CAS) inhibited THP-1 cell adherence to activated BEAS-2B cells. **(A)** CAS decreased the levels of ICAM-1 in BEAS-2B cells activated with TNF-α. The data are presented as means ± SEM of three independent experiments (*n* = 9). ^∗^*p* < 0.05, ^∗∗^*p* < 0.01 compared to BEAS-2B cells stimulated with TNF-α. **(B)** Fluorescence microscope images of THP-1 cells labeled with calcein-AM and mixed with untreated cells (normal control, N) and TNF-α-activated BEAS-2B cells in the absence or presence of CAS.

Enzyme-linked immunosorbent assay showed that casticin can inhibit ICAM-1 production in TNF-α-stimulated BEAS-2B cells (**Figure 8A**). Monocyte THP-1 cells stained with calcein-AM and co-cultured with TNF-α-stimulated BEAS-2B cells demonstrated that casticin prevented THP-1 cell adhesion to BEAS-2B cells (**Figure 8B**).

### Effect of Casticin on ROS Production

**FIGURE 9:**
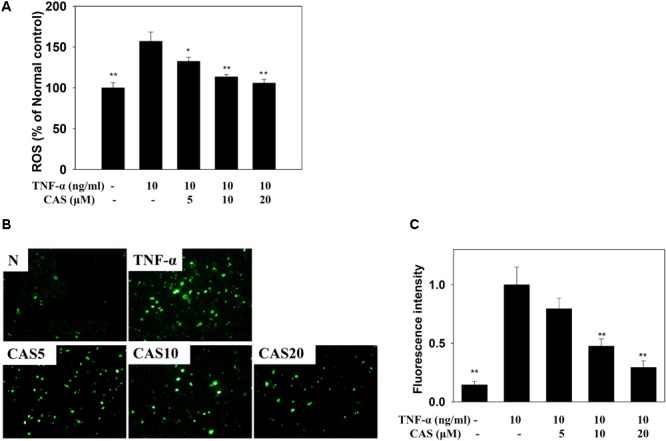
Effects of casticin (CAS) on reactive oxygen species (ROS) production in activated BEAS-2B cells. **(A)** Percentage of ROS detected in TNF-α-activated BEAS-2B cells in the absence or presence of CAS compared to untreated cells (normal control, N). The ROS levels of untreated cells were indicated as 100% **(B)**. Fluorescence microscope images of intracellular ROS. **(C)** Fluorescence intensity of intracellular ROS. The data are presented as means ± SEM of three independent experiments (*n* = 9). ^∗^*p* < 0.05, ^∗∗^*p* < 0.01 compared to BEAS-2B cells stimulated with TNF-α alone.

Casticin decreased ROS production in TNF-α-stimulated BEAS-2B cells treated with DCFH-DA (**Figure 9A**). In addition, fluorescence microscopy showed that casticin suppressed intracellular ROS compared to TNF-α-stimulated BEAS-2B cells (**Figures 9B,C**).

## Discussion

In Chinese traditional medicine, the fruit of *V. trifolia* and *V. rotundifolia* are common medical herbs for treating headaches, dizziness, bronchitis, gingival swelling, colds, and inflammation (Ono et al., 2001; Matsui et al., 2009). Casticin is a major flavonoid of *Vitex fructus, V. trifolia*, and *V. rotundifolia*, and has been found to inhibit the proliferation of cancer cells and induce apoptosis in some cancers, including gastric cancer, lung cancer, oral cancer, cervical cancer, glioma, and hepatocellular carcinoma (He et al., 2014; Rasul et al., 2014; Lee et al., 2015; Liou and Huang, 2017; Chou et al., 2018). Other studies have found that casticin improves liver fibrosis by suppressing TGF-β and the Smad pathway, and it may also protect against the inflammatory response in LPS-induced acute lung injury in mice by inhibiting the NF-κB and NLRP3 pathways (Wang et al., 2016; Zhou et al., 2017). A previous study also found that casticin can suppress pro-inflammatory cytokine production in LPS-stimulated macrophages or IL-1β-activated lung epithelial cells (Liou et al., 2014; Liou and Huang, 2017). In the current study, casticin significantly reduced AHR, tracheal goblet cell hyperplasia, and eosinophil infiltration in the lungs of asthmatic mice. Casticin also modulated inflammatory mediators, Th2-associated cytokines and chemokines in BALF and lung tissue, and attenuated oxidative stress in lung tissue. Furthermore, casticin clearly weakened OVA-IgG1 and OVA-IgE in serum, and reduced Th2 cytokine levels in OVA-stimulated splenocytes. Moreover, casticin decreased the levels of pro-inflammatory cytokines and chemokines and ROS expression in inflammatory human bronchial lung epithelial (BEAS-2B) cells, and reduced ICAM-1 expression in BEAS-2B cells, blocking THP-1 adhesion. These results suggest that casticin improves asthma symptoms by alleviating the inflammatory response, oxidative stress, and Th2 cell activity in this murine model of OVA-induced asthma.

Acute asthma attacks not only result in rapid short breaths, but also excessive mucus and an obstructed airway, causing dyspnea and death (Bush et al., 2017). In the airways of asthma patients, allergens could stimulate the hyperplasia and activation of goblet cells to secret excessive mucus (Sastre et al., 2017). Previous studies have found that IL-13 could contribute to the promotion of goblet cell proliferation, and IL-13 knockout mice with asthma do not exhibit promoted goblet cell hyperplasia in the airways or Muc5AC expression in lung tissue (Chen et al., 2014; Sun et al., 2017; Zhao et al., 2017). In **Figures 2, 4**, our experiments show that casticin had the ability to reduce goblet cell hyperplasia and reduce Muc5AC and gob5 gene expression in asthmatic mice. Thus, casticin could contribute to improving mucus hypersecretion for decreased dyspnea. Moreover, IL-13 is considered to induce and increase AHR, causing airway narrowing and exaggerated bronchoconstriction. Interestingly, asthmatic mice treated with IL-13 via injection into the trachea have significantly increased AHR, and IL-13 knockout fails to promote AHR in asthmatic mice (Eiymo Mwa Mpollo et al., 2016). As, casticin significantly reduced IL-13 levels in BALF and decreased IL-13 gene expression in the lungs of asthmatic mice, our findings confirm that casticin reduced AHR by reducing IL-13 expression, improving shortness of breath and lung function.

Previous studies have shown that allergen-stimulated airways activate immune cells to release inflammatory cytokines, including IL-6 and TNF-α, which not only increase inflammation in the lungs, but also contribute to exacerbation of pathological changes in the lungs, including pulmonary cell damage and fibrosis (Ball et al., 2016; Leomicronn, 2017). TNF-α also induces an inflammatory response in the tracheal epithelium to release more inflammatory cytokines that exacerbate lung cell damage, and the expression of more chemokines to attract immune cells to migrate into allergic lung tissue in asthmatic mice (Desai and Brightling, 2010; Sun et al., 2017). We found that casticin not only reduced IL-6 and TNF-α expression in BALF, but also decreased IL-6, IL-8, MCP-1, and CCL5 production in TNF-α-stimulated BEAS-2B cells. Therefore, casticin could attenuate the inflammatory response and reduce immune cell infiltration into lung tissue in asthmatic mice. In **Figures 4, 5**, analysis of gene expression in the lungs also found that casticin could reduce inflammatory mediators, including IL-6, iNOS, and COX-2, with reductions in iNOS and COX-2 protein expression in asthmatic mice. Thus, casticin significantly reduced inflammatory mediators, ameliorating lung damage in asthma. However, it is not easy to induce BEAS-2B cells to express eotaxin using TNF-α, and previous experiments showed that TNF/IL-4 could stimulate BEAS-2B cells to express eotaxin, which attracted eosinophils into allergic airways and lung tissue in asthma (Huang et al., 2014). We found that casticin could inhibit the levels of CCL11, CCL24, and CCL26 in TNF-α/IL-4-activated BEAS-2B cells and decrease CCL11 and CCL24 production in the BALF and lungs of asthmatic mice. These results confirmed that casticin had the ability to reduce eotaxin production by tracheal epithelial cells, blocking eosinophil migration into the lung tissue of asthmatic mice. In addition, many studies have shown that asthmatic lungs activate Th2 cells, and that activated Th2 cells secrete excess IL-5 to induce bone marrow cell differentiation, activating eosinophils to infiltrate the lung tissue (McBrien and Menzies-Gow, 2017). Our experiment found that casticin has the ability to reduce the levels of IL-5 for suppressing eosinophil infiltration, reducing inflammation and the allergic response in the lungs of asthmatic mice.

Inflammatory tracheal epithelial cells express an adhesion factor, ICAM-1, that adheres to chemotactic immune cells in the airways and lungs (Lambrecht and Hammad, 2012). In **Figures 1, 4**, animal experiments have found that OVA-sensitized mice contain high levels of immune cells in BALF and lung tissue, and casticin could decrease ICAM-1 expression in the lungs and BALF. In *in vitro* assays, casticin significantly decreased THP-1 cell adherence to BEAS-2B cells. Therefore, casticin can be speculated to have reduced immune cell infiltration in the lungs through decreased ICAM-1 expression.

Allergic asthma is a complex inflammatory disease associated with airway remodeling and AHR (Tanno et al., 2017). Many studies have indicated that Th2 cells secrete excessive IL-4 in asthmatic patients, and IL-4 would induce B cell activation to produce more IgE (Leomicronn, 2017; McBrien and Menzies-Gow, 2017). Therefore, high IgE levels are found in the blood of asthma patients. Activated eosinophils could express IgE𝜀RI receptor to bind with IgE, and the activated eosinophils could release excessive inflammatory and allergic mediators, causing the repeated attacks of allergic and inflammatory reactions in the lungs (McBrien and Menzies-Gow, 2017). In **Figure 6**, we found that asthmatic mice treated with casticin not only had the ability to suppress IL-4 levels in BALF and the lungs, but also had reduced serum levels of OVA-IgE and OVA-IgG1. Moreover, casticin significantly decreased Th2-associated cytokines in OVA-stimulated splenocytes. Therefore, casticin can be speculated to have inhibited the development of asthma symptoms by reducing Th2 cell activity in asthmatic mice.

Excess ROS could aggravate more inflammatory immune cells to infiltrate the lung tissue, stimulating airway epithelial cells to secret more inflammatory cytokines (Nesi et al., 2017b). Many studies have pointed out that oxidative stress would induce aggravated bronchospasms and a serious inflammatory response, deteriorating lung damage in asthma patients (Araujo et al., 2012; Nesi et al., 2017a). Natural antioxidants resveratrol and quercetin have been shown to improve the pathological manifestations of asthma by blocking oxidative stress in asthmatic mice (Wood et al., 2010; Lekshmi et al., 2015). Casticin has anti-oxidative effects, blocking the inflammatory response in LPS-stimulated macrophages by promoting the expression of antioxidant protein HO-1 (Liou et al., 2014). Casticin could also improve oxidative stress in the inflammatory lung by regulating myeloperoxidase activity in LPS-induced acute lung injury (Wang et al., 2016). In the current study, casticin could significantly increase the expression of Nrf2 and HO-1 in lung tissue from asthmatic mice, improving oxidative stress. Moreover, GSH could provide antioxidant protection, decreasing the development of chronic inflammatory asthma (Chan et al., 2017). Lipid metabolism and prostaglandin biosynthesis would increase MDA, a lipid peroxidation marker, and increase oxidative stress, inducing aldehyde metabolites and the release of toxins that cause tissue damage (Celik et al., 2012; Nesi et al., 2017b). In **Figure 5**, the results demonstrated that casticin significantly contributes to increased GSH levels and reduced MDA expression, which attenuated oxidative stress in the lungs of asthmatic mice. Moreover, casticin significantly reduced ROS expression in TNF-α-stimulated BEAS-2B cells. Thus, casticin had a good anti-oxidative protective effect to ameliorate lung damage through the regulation of ROS in asthma.

## Conclusion

The present study demonstrated that casticin can significantly reduce inflammatory and oxidative stress, ameliorating AHR, mucus hypersecretion, and eosinophil infiltration, by suppressing Th2 cell activity in asthmatic mice. Therefore, casticin has potential for treating and regulating inflammation and anti-oxidative stress in asthma.

## Author Contributions

C-JL, C-YC, Y-HW, and W-CH designed and performed the experiments. C-JL, K-WY, and C-YC analysis and interpretation of data. C-JL, Y-HW, and W-CH drafting the manuscript.

## Conflict of Interest Statement

The authors declare that the research was conducted in the absence of any commercial or financial relationships that could be construed as a potential conflict of interest.
